# Wnt/Β-Catenin and Sex Hormone Signaling In Endometrial Homeostasis and Cancer

**DOI:** 10.18632/oncotarget.201

**Published:** 2010-10-12

**Authors:** Yongyi Wang, Marten van der Zee, Riccardo Fodde, Leen J Blok

**Affiliations:** ^1^ Department of Obstetrics & Gynaecology, Josephine Nefkens Institute, Erasmus University Medical Center Rotterdam, PO Box 2040, 3000 CA Rotterdam, The Netherlands; ^2^ Departments of Pathology, Josephine Nefkens Institute, Erasmus University Medical Center Rotterdam, PO Box 2040, 3000 CA Rotterdam, The Netherlands

**Keywords:** Wnt/β-catenin, estradiol, progesterone, endometrium

## Abstract

A delicate balance between estrogen and progestagen signaling underlies proper functioning of the female reproductive tract and, in particular, the monthly re- and degenerative phases characteristic of the menstrual cycle. Here, we propose that the canonical Wnt/β-catenin signaling pathway may underlie this finely tuned hormonal equilibrium in endometrial homeostasis and, upon its constitutive activation, lead to neoplastic transformation of the endometrium. During the menstrual cycle, estradiol will enhance Wnt/β-catenin signaling in the proliferative phase, while progesterone inhibits Wnt/β-catenin signaling, thus restraining estrogens' proliferative actions, during the secretory phase. In case of enhanced or unopposed estrogen signaling, constitutive activation of Wnt/β-catenin signaling will trigger endometrial hyperplasia, which may develop further into endometrial cancer.

## SEX HORMONE SIGNALING IN THE ENDOMETRIUM

The inner layer of the human uterus, the endometrium is a dynamic tissue that undergoes hundreds of cycles of proliferation, differentiation and shedding during a woman's reproductive years [1]. The endometrium can be divided into two layers, the *functionalis* and the *basalis*. Whereas the *functionalis* layer comprises the upper two-thirds of the endometrium and is shedded during menstruation, the *basalis* includes the lower one-third which remains intact and is responsible for producing a new *functionalis* during each subsequent menstrual cycle [1] [2].

It is the fine balance between the activities of the two female sex hormones, estradiol and progesterone, which determines lineage faith in the endometrium. During the first two weeks of the menstrual cycle the thecal cells of the ovary produce large amounts of estradiol. Simultaneously, endometrial estrogen receptor (ER) levels (mainly ERα) are increased [3]. Upon ligand binding, ER dimers will translocate to the nucleus where they activate transcription of downstream target genes (e.g. insulin like growth factor; *IGF-1*), that stimulate endometrial proliferation [4]. Later, during the third and fourth week of the menstrual cycle, the corpus luteum starts producing progesterone thus inhibiting estradiol-induced proliferation of endometrial cells and stimulating cellular differentiation [5] [6]. If no fertilized oocytes are implanted in the uterus, the corpus luteum cannot be maintained due to lack of human chorion gonadotropine (HCG), and both estrogen and progesterone levels will decline. Withdrawal of progesterone leads to endometrial cell apoptosis and tissue breakdown of the *functionalis* of the endometrium, resulting in its shedding from the uterus [7].

The inhibition of estrogens' mitotic activity exerted by progesterone is of clinical relevance as unopposed or increased estrogen action in women is a well-established risk factor for endometrial cancer [8] [9].

### Unbalanced sex hormone signaling can induce endometrial cancer

Endometrial cancer is one of the most common cancers of the female genital tract [10]. It accounts each year for approximately 142.000 new cases diagnosed worldwide, and for 42.000 deaths [11] [12]. Endometrial cancer is the seventh most common malignant disorder and its incidence is expected to increase in the near future due to the increase in life span expectancy and obesity [13].

Based on epidemiology, conventional histopathology, and clinical behavior, endometrial carcinoma can be divided into two subtypes. Type I endometrial cancer, comprising approximately 85% of the total endometrial carcinoma burden among western societies, resembles normal endometrial hyperplasia in morphology and is associated with increased or unopposed estrogen signaling [12]. Type I endometrial cancer often shows mutations in the *PTEN* and in DNA mismatch repair genes (*MLH1*, *MSH2*, *MSH6*). Also, oncogenic mutations in *KRAS* and/or *CTNNB1* (β-catenin) are recognized major alterations [14] [15] [16]. Type II endometrial cancer occurs predominantly in older post-menopausal women, is not correlated to increased estrogen exposure, and is generally associated with a poorer prognosis. Type II endometrial cancers often show mutations in *TP53* and *ERBB2* (Her-2/neu) [12] [15] [16].

In western, industrialized countries, two large groups of women are at increased risk of developing endometrial cancer: (i) women with significant overweight [13], and (ii) those receiving tamoxifen for breast cancer treatment [17]. Tamoxifen is a selective estrogen receptor modulator (SERM) acting as an antiestrogen in mammary tissue, but showing estrogenic activity in the endometrium [18]. Currently, it is estimated that up to 40% of all endometrial cancers could be related to obesity [13]. Since the prevalence of obesity is increasing, the incidence of obesity-related endometrial cancer is also on the rise [19]. Additional risk factors for the development of endometrial cancer include: polycystic ovarian syndrome, skipping menstrual periods, being nulliparous, late menopause onset, and the use of unopposed estrogen as hormone substitution therapy [20]. Hence, enhanced estrogen signaling for prolonged periods of time, also as the result of insufficient progesterone levels, represents the main risk factor in endometrial cancer.

Progesterone antagonizes the proliferative activity of estrogen by inducing epithelial and stromal cell differentiation in the endometrium [21] [22]. In fact, although progesterone (in the form of medroxyprogesterone acetate (MPA)) can be used for the palliative treatment of well-advanced and recurrent endometrial cancer, this treatment has a modest response rate (15-25%) [23]. In contrast, when progesterone, is employed as a primary treatment (e.g. in pre-menopausal women suffering from well-differentiated endometrial cancer and determined to preserve fertility), response rates are considerably improved (up to 60% or more) [24] [25].

### Progesterone inhibition of estrogen-induced proliferation

The mechanisms by which progesterone induces its anti-proliferative effect on the endometrium are still largely unknown. Several studies have shown a direct effect of progesterone on estrogen receptor signaling and expression levels [26], while others show that progesterone affects the availability of bioactive estrogens [27] [28] [29]. Furthermore, differential expression of the progesterone receptor (PR) isoforms (PRA and PRB) and the relative expression levels of ERα compared to ERβ could also play a role in controlling endometrial proliferation during the menstrual cycle [30] [27].

**Figure 1:**
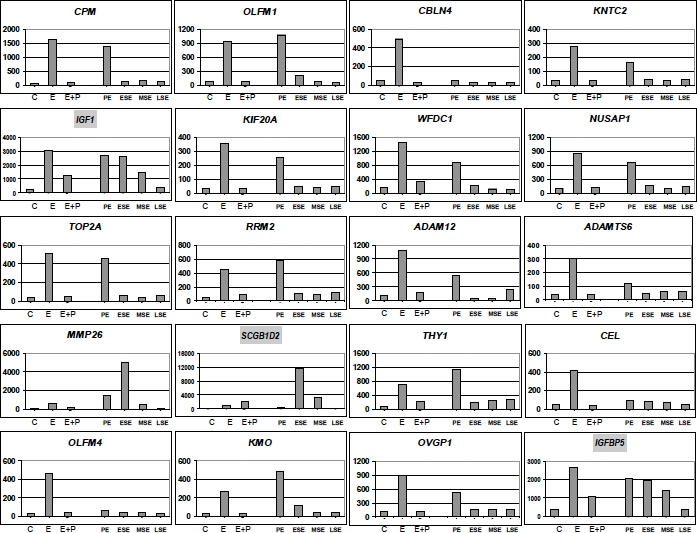
Inhibition of estrogen-induced genes by progesterone The data employed in this figure were obtained by combining endometrial gene expression profiles from postmenopausal women after no treatment (C), 21-day treatment with estradiol (E), or estradiol + medroxyprogesterone acetate (E+P) [22], with gene expression profiles from the proliferative endometrium (PE), early secretory endometrium (ESE), mid secretory endometrium (MSE), or late secretory endometrium (LSE) [4]. The bars represent the relative expression levels of the indicated genes under the indicated conditions. For this figure the twenty most significantly estrogen regulated genes are shown. The three highlighted gene-names are those discussed in depth in the text.

In order to investigate in more detail the molecular mechanism(s) underlying progesterone-driven inhibition of estrogen signaling, expression profiling analysis was conducted on endometrial tissue specimens obtained from postmenopausal women treated for three weeks with estrogens (E-only) or estrogens+progestagens (E+P) and compared to untreated controls [21] [22]. The endometrial gene profiles obtained from this study were also compared with endometrial gene expression levels of untreated women during the different stages of the menstrual cycle [4]. Upon reviewing regulation of expression of the twenty most significantly estrogen-regulated genes (Fig. 1), it was observed that most of them were completely counterbalanced by concurrent administration of progestagens (E+P). However, estrogen-induced upregulation of specific genes (e.g. *IGF1* and *IGFBP5*) is only partly counterbalanced by progestagens, whereas in other cases (e.g. *SCGB1D2*) the increase of expression level upon estrogen signaling is not at all counterbalanced by progestagens. Notably, these genes also behave differently during the menstrual cycle: *IGF1* and *IGFB5* are upregulated during the proliferative, early and mid secretory phases of the menstrual cycle, whereas *SCGB1D2* is upregulated exclusively during the early and mid secretory phases (Fig. 1).

Next to gene profiling studies, immunohistochemical analysis of PRA/PRB and ERα expression were performed together with measurements of estrogen availability [21]. These investigations revealed that besides a small increase in PRA/PRB levels in glandular cells upon treatment with E-only, no pronounced differences in ERα and PR expression levels were observed between the E-only and the E+P group [21]. Furthermore, in order to fully asses estrogen signaling, next to the biologically most potent estrogen, estradiol (E_2_), the less active estradiol precursor, estrone (E1), as well as its inactivated form, sulfated estrone (E1S), were measured [28] [31] [32]. It was observed that both in sera and in uterine tissues, estradiol, estrone and sulfated estrone were increased as a result of E-only and E+P treatment, though with no significant difference between the treatment groups [32] These studies seem to indicate that progestagenic regulation of estrogen signaling at the receptor level and at the level of ligand availability is not the major mechanism through which progesterone counterbalances estrogen signaling during the menstrual cycle, and additional molecular mechanisms are likely to play a role.

In literature there are a number of reports implicating an important role for Wnt/β-catenin signaling in regulating endometrial proliferation and differentiation. For example, the activity of Wnt/β-catenin signaling was observed to change between different stages of the menstrual cycle [33] and plays an important role in preparing the endometrium for embryo implantation [34] and subsequent placental formation [35]. Furthermore, Wnt/β-catenin signaling is often found activated in endometrial cancer [14] [36] and, upon reviewing regulation of estrogen and progesterone regulated genes [21] [22] [4], it was also observed that a significant number of downstream targets and components of the Wnt/β-catenin signaling pathway were regulated [37]. Moreover, some of the sex hormone-regulated downstream targets and components of the Wnt/β-catenin signaling pathway have also been implicated in endometrial carcinogenesis: both *FOXO1* and *CDH1* (E-cadherin) expression is decreased in endometrial cancer and is induced by progesterone [38] [39] while Survivin (*BIRC5*) is expressed at high levels in endometrial cancer and upregulated by estradiol [40]. Based on these observations, a role for Wnt/β-catenin signaling downstream from sex hormone signaling in the endometrium was hypothesized and will be discussed in more detail in the next paragraphs.

## THE RELATIONSHIP BETWEEN SEX HORMONE AND WNT/Β-CATENIN SIGNALING IN THE ENDOMETRIUM

Central in canonical Wnt signaling is the destruction complex, a multi-protein complex consisting of the scaffold proteins AXIN1 and AXIN2 (conductin), β-catenin (CTNNB1), the tumor suppressor APC (adenomatosis polyposis coli) and the Ser-Thr kinases CK1 (casein kinase I) and GSK3β (glycogen synthase kinase 3 beta) [41]. In the absence of Wnt ligands, formation of the destruction complex triggers Thr/Ser-phosphorylation of β-catenin by CK1 and GSK3β, and its subsequent ubiquitination and proteasomal degradation. Upon Wnt signaling, the formation of the destruction complex is inhibited thus leading to cytoplasmic accumulation of β-catenin and its nuclear translocation [42]. Once in the nucleus, β-catenin interacts with members of the TCF/LEF transcription factor family, thus regulating the expression of a broad spectrum of Wnt downstream target genes [42] [43] [44]. The latter include genes encoding for proteins with a central role in cell proliferation and survival such as cyclin D1 (*CCND1*) and *c-MYC*, in addition to a broad spectrum of other cellular functions i.e. cellular migration (e.g. *CD44*), cell adhesion (*CDH1*), extracellular matrix (*MMP7*) and many others [42] [45].

The central role of Wnt/β-catenin signaling in the regulation of tissue homeostasis has been extensively investigated for the gut [42] [46]. Along the intestinal tract, stem cells are located at the bottom of the crypts of Lieberkuhn where they give rise to new stem cells and to proliferating progenitor cells (transient amplifying, TA, cells) [47]. These progenitor cells actively divide and produce new cells that are pushed up along the flank of the crypt towards the villus and eventually differentiate into Goblet cells, enteroendocrine cells and absorptive epithelial cells [48]. A somewhat similar process seems to take place in the endometrium, where estrogen receptor activation in the *basalis* stimulates endometrial cell proliferation during the first two weeks of the menstrual cycle, thus giving rise to the *functionalis*. In week three and four of the menstrual cycle, the corpus luteum will produce progesterone, which reduces estrogens-driven proliferation and induces differentiation of the *functionalis* thus preparing the endometrium for implantation of the fertilized oocyte around day 20 - 22 of the menstrual cycle.

Wnt/β-catenin signaling activity along the crypt-villus axis of the intestine follows a decreasing gradient from the stem cell (SC) and proliferative (TA) compartment to the more differentiated compartment [49]. In the endometrium Wnt/β-catenin signaling has also been suggested to play a role in regulating proliferation and differentiation during the menstrual cycle. Nei et al. [33] observed that in the human endometrium, nuclear β-catenin was clearly enhanced during the proliferative phase of the menstrual cycle, while it was mostly found in the cytoplasm and at the cell membrane during the secretory phase. Recently, it was also observed that estradiol induces stabilization of intracellular β-catenin in the endometrium, and, upon inhibition of Wnt signaling (by using adenoviral *SFRP2*), estradiol-induced proliferation was abolished [50]. In two other studies [51] [52], LiCl was administered through the drinking water of mice to inhibit GSK3β activity and thereby activate Wnt signaling. LiCl-treated animals were characterized by increased proliferation and hyperplasia of the endometrium, thus mimicking sustained estrogen signaling.

In summary, it seems likely that, during the menstrual cycle, sex hormones can modulate Wnt/β-catenin signaling to maintain the balance between proliferation and differentiation.

### Estradiol regulation of Wnt/β-catenin signaling

The putative mechanisms underlying estrogen-mediated Wnt/β-catenin activation in the uterus are at present poorly understood. A direct effect of ERα as a transcription factor on the expression of Wnt ligands, modulators and targets has been described by many authors: the ligands Wnt4, Wnt5A and Wnt7A have been shown to be induced by estradiol [53] [54] [50] [55] [56]; the Wnt inhibitor DKK1 was shown to be inhibited by estrogens in bone forming osteoblasts [57] and in the hippocampal CA1 region [58] and last, the Wnt-target gene *WISP2* (Wnt-1 induced signaling pathway protein 2) was shown to be upregulated through direct interaction of activated ERα with its promoter region in human breast cancer cells [59].

ERα, however, can also function as a transcriptional modulator without directly binding to DNA sequences in the promoter region of the affected genes. For example, Shi et al [60] could show that EZH2 (the polycomb group protein enhancer of zeste homolog 2) physically interacts with ERα and β-catenin. Hence, estradiol can affect the transcription of Wnt/β-catenin target genes without directly interacting with estrogen response elements at the DNA level.

**Figure 2:**
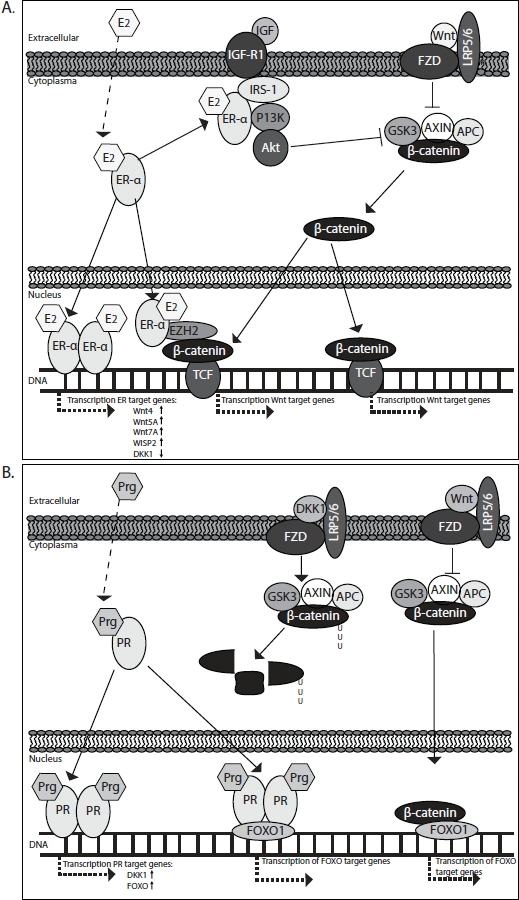
Estrogen (A) and progestagen (B) regulation of Wnt/β-catenin signaling in the endometrium

**Figure 3:**
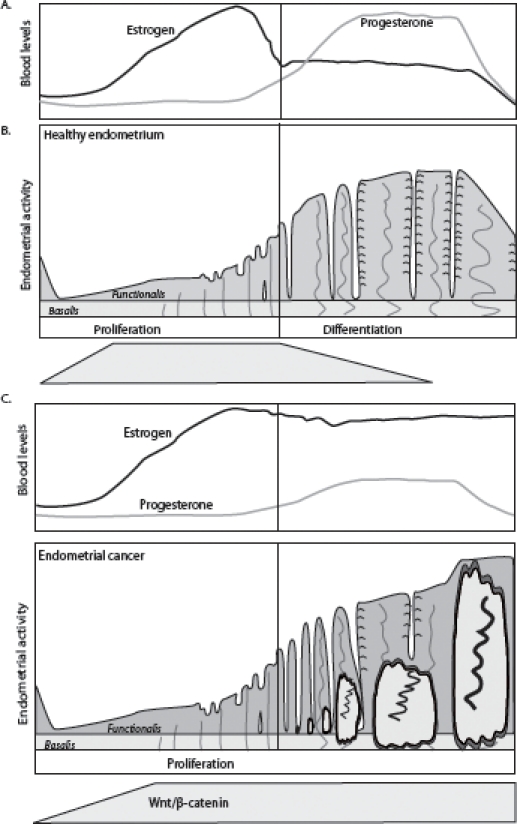
Hormonal and morphological changes during the normal menstrual cycle (A) and in case of increased estrogen activity (B) In case of enhanced or unopposed estrogen signaling, the endometrium may develop hyperplasia, which may eventually develop further into endometrial cancer.

Furthermore, ERα has also been observed to associate with important growth factor pathways such as the PI3K pathway thus indirectly cross-talking with canonical Wnt signaling [61] [62]. Binding of ERα to p85 (PIK3R2), the regulatory subunit of PI3K, activates AKT1 which results in inhibition of GSK-3β. This, in turn, prevents N-terminal Ser/Thr phosphorylation of β-catenin, enhances its intracellular stabilization and eventual allowes for translocation to the nucleus, where it complexes with members of the TCF/LEF transcription factor family and lead to the activation of Wnt target genes [63] [64] (Fig. 2A and Fig 3).

### Progesterone regulation of Wnt/β-catenin signaling

A role for progesterone in regulating Wnt/β-catenin signaling was suggested by Kao et al. [65] and by Tulac et al. [66] who observed a profound progesterone-specific increase in expression of the Wnt/β-catenin signaling inhibitor *DKK1* in endometrial stroma cells during the secretory phase of the menstrual cycle. Using the antiprogestagen mefipristone (RU486), Catalano et al. [67] could indeed confirm progestagen regulation of many Wnt/β-catenin pathway components. Furthermore, Kane at al. [68] subsequently showed that TGFβ1 attenuates both the expression of *PR* and *DKK1* in differentiated endometrial stromal cells corroborating a close link between progesterone and Wnt/β-catenin signaling.

Recently, our group has followed up on these observations [37] and could show that progesterone efficiently inhibited the expression of a Wnt/β-catenin signaling reporter plasmid (TOP/FOPflash) in the Ishikawa endometrial cancer cell line by induction of the Wnt/β-catenin inhibitors *DKK1* [66] and *FOXO1* [38]. Furthermore, when induction of both *DKK1* and *FOXO1* was prevented, progesterone inhibition of Wnt signaling was also partly circumvented. FOXO1, in this respect, is an interesting molecule, as it has been shown to physically interact with the progesterone receptor to coordinate cell cycle regulation and differentiation of human endometrial stromal cells [69]. Furthermore, FOXO1 is also able to interact with β-catenin [70] [71], thus possibly directly inhibiting Wnt/β-catenin signaling [72].

Another pathway likely to play a role in regulating the interplay between progesterone and Wnt/β-catenin signaling is Hedgehog (Hh). Combined endometrial microarray data from different stages of the menstrual cycle [4] and from E-only or E+P treated patients [22] indicated profound sex hormone regulation of Hh signaling. The Hedgehog ligand *IHH* (Indian hedgehog), its receptor *PTCH*, and the transcription factor and target gene *GLI1* are all up-regulated upon estrogen signaling and downregulated by progesterone during the menstrual cycle. Furthermore, it has been shown that when *IHH* expression is impaired, the downstream effects of progesterone are lost in the uterus [73]. As indicated, progesterone itself downregulates Hh signaling in the uterus and it has been shown that Wnt/β-catenin signaling may act downstream to Indian hedgehog signaling [74].

Recently the link between Hedgehog and Wnt/β-catenin signaling has also been confirmed for atypical endometrial hyperplasia and endometrial cancer: in hyperplasia and in well differentiated endometrial cancers GLI1 overexpression overlaps with β-catenin nuclear immunoreactivity [75]. Because our own data on staining for the Wnt target gene CD44 indicated that progesterone can also act as a profound inhibitor of Wnt/β-catenin signaling *in vivo* in hyperplasia as well as in well differentiated endometrial cancer, a physiological and functional link between progesterone, hedgehog and Wnt/β-catenin signaling seems plausible. (Fig. 2B and Fig. 3)

## ROLE OF WNT SIGNALING IN ENDOMETRIAL CANCER

**Figure 4:**
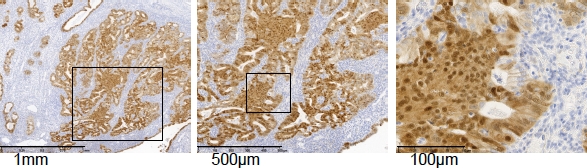
Nuclear β-catenin accumulation in well differentiated endometrial cancer Immunohistochemical staining for β-catenin of type I endometrial cancer showing areas with nuclear β-catenin accumulation.

Gene mutations leading to constitutive activation of canonical Wnt signaling have been found in many different cancer types (e.g. breast, colon, stomach, liver, ovary skin etc). Also in the case of endometrial cancer, activation of Wnt/β-catenin signaling is likely to play an important role in early tumorigenesis [14] [36]. A substantial fraction of well differentiated endometriod carcinomas (Type I) cases (31%: [76]; 85%: [77]) show nuclear β-catenin staining (Fig. 4). Accordingly, loss- and gain-of-function mutations in members of the Wnt/β-catenin signaling pathway known to act as tumor suppressors (APC) and oncogenes (β-catenin) have also been identified. β-catenin activating mutations at its GSK-3β binding consensus site located within exon 3 have been identified in 15-40 % of endometrial tumors [78] [79], whereas LOH at the *APC* locus was observed in 24 % of the cases with nuclear β-catenin staining [80]. *APC* mutation analysis showed truncating mutations in 10% of all endometrial cancers [81]. Moreover, the *APC* A1 promoter was found to be hypermethylated in 46.6% of endometrial cancers with nuclear β-catenin [80], often in correlation with micro-satellite instability [82]. Notably, these somatic mutations in members of the canonical Wnt pathway were preferentially found in Type I endometrial cancer, which accounts for about 85% of the total number of endometrial cancer cases. Enhanced estrogen signaling over a prolonged period of time is believed to be a causative factor for Type I endometrial cancer [9]. Hence, although continuous estrogen induced Wnt/β-catenin signaling may represent an early step in endometrial tumorigenesis, tumor progression and malignant transformation seem to additionally require somatic mutations leading to the constitutive activation of the pathway (Fig. 3).

### Current investigations into the role of Wnt/β-catenin signaling in endometrial carcinogenesis

Enhanced or unopposed estrogen signaling is the most important risk factor for endometrial hyperplasia and endometrial cancer. In view of the observations according to which i. Wnt/β-catenin signaling plays a central role in endometrial homeostasis, ii. it possibly represents one of the downstream effectors of estrogen signaling, and iii. its constitutive activation is likely to underlie malignant transformation in the uterus, it is important to assess whether canonical Wnt can trigger endometrial hyperplasia and cancer in the absence of enhanced estrogen signaling. In other words, among the allegedly broad spectrum of downstream effectors of estrogen signaling, does constitutive Wnt/β-catenin activation play a rate-limiting role for endometrial malignancies?

To this aim, several genetically engineered mouse models have been employed. Tanwar et al. [83] investigated the effects of conditional Wnt signaling activation in the uterus by *Amhr2-Cre*-driven oncogenic activation of β-catenin (*Ctnnb1^tm1Mmt/+^*). The *Amhr2* gene is expressed as of embryonic day 12.5 onwards in mesenchymal cells surrounding the Mullerian duct and in adult mice in the myometrium. The corresponding *Amhr2^Cre/+^;Ctnnb1^tm1Mmt/+^* mice develop myometrial hyperplasia and mesenchymal tumors (similar to leiomyomas) and endometrial sarcomas. Furthermore, hyperplasia of endometrial glands was occasionally observed in the uterus, suggesting that mesenchymal activation of Wnt/β-catenin signaling plays a role in the early events of epithelial tumorigenesis in the endometrium. Jeong et al. [84] employed *Pgr-Cre* to drive oncogenic activation of β-catenin (*Pgr^Cre/+^;Ctnnb1^f(Ex3)/+^*) and of canonical Wnt signaling in a broad spectrum of uterine cells (endometrium + myometrium). These authors could show that activation of Wnt/β-catenin signaling in the uterus resulted in enhanced proliferation of glandular epithelial cells, endometrial hyperplasia at 6 weeks of age, and in defective estrogen signaling, though not in endometrial cancer. These results suggest that constitutive activation of Wnt/β-catenin signaling on its own is insufficient for endometrial cancer onset and possibly that the synergistic action of additional downstream effectors of estrogen signaling are necessary for full-blown malignant transformation. However, as predicted by the ”just-right” signaling model [85] [86], different levels of pathway activation may differently trigger tumorigenesis in distinct tissues. Therefore, it would be of interest to investigate the consequences of hypomorphic mutations in members of the Wnt pathway other than β-catenin whose oncogenic activation invariably leads to extremely high Wnt signaling levels. By inducing *Apc* mutations in the myometrium [87], we recently observed defects in the myometrial layer of the uterus, where a significant loss of muscle fibers was apparent [Wang et al, submitted). Likewise, we also employed *Pgr-Cre* to drive loss of *Apc* function in epithelial cells of the endometrium and in myometrial cells. Also in this case, muscular defects were apparent with invasion of endometrial glands and stroma into the muscular layer. Notably, we also observed hyperplasia and early stage endometrial cancer [manuscript in preparation). Hence, it is possible that “just-right” levels of Wnt/β-catenin signaling are sufficient to trigger tumor initiation in the endometrium in a dosage- and context-dependent fashion.

## CONCLUSIONS

In this review we discussed the role of estrogens and progestagens in the regulation of Wnt/β-catenin signaling as well as the involvement of activated Wnt/β-catenin signaling in the development of endometrial cancer. Evidence indicates that estrogens can induce Wnt/β-catenin signaling and that enhanced or unopposed estrogen signaling, as well as activated Wnt/β-catenin signaling may underlie endometrial hyperplasia and cancer. Furthermore, progesterone was shown to be a strong inhibitor of Wnt/β-catenin signaling. The latter is of clinical relevance as it is well known that progesterone can inhibit endometrial hyperplasia and well differentiated endometrial cancer growth.

These observations place Wnt/β-catenin signaling at the center of physiological regulation of the menstrual cycle and point at a central functional role for its uncontrolled activation in endometrial carcinogenesis. Although Wnt/β-catenin signaling inhibitors (e.g. CGP049090, PKF115-584 [88] and XAV939 [89]) have not yet been introduced into clinical practice and likewise have not been considered for endometrial cancer treatment, the experimental data here summarized may lay the basis for future tailor-made therapies based on members of the canonical Wnt pathway or its downstream targets.
